# Genomic and clinical determinants of outcome in Irish AML: a real-world analysis (2007–2025), single centre experience

**DOI:** 10.1007/s00277-026-07081-2

**Published:** 2026-05-30

**Authors:** Ezzat Elhassadi, Ellen O’Rourke, Ahmed Bannaga, Senthil Kumar, Brain Hennessy

**Affiliations:** Department of Haematology, Royal College Of Surgeons Ireland, University Hospital Waterford, Waterford, Ireland

**Keywords:** AML, Karyotyping, Molecular, ELN risk stratification, Treatment outcome

## Abstract

Acute myeloid leukaemia (AML) is a genetically heterogeneous malignancy in which cytogenetic and molecular features critically inform diagnosis, prognosis, and therapeutic decision-making. While traditional karyotyping has long guided risk stratification, high-throughput sequencing has revealed extensive molecular diversity, particularly within cytogenetically normal AML, reshaping AML classification and management. This study characterises the genomic landscape and clinical outcomes of an Irish AML cohort using integrated cytogenetic and molecular profiling. We retrospectively analysed 166 adult AML patients diagnosed at our cancer centre between 2007 and 2025. Demographic, clinical, cytogenetic, molecular, and treatment data were collected. Karyotype-based and ELN 2022/2024 molecular classifications were applied. Survival analyses were performed using Prism v10.06, with statistical significance assessed using the log-rank (Mantel–Cox) test. Mutation frequencies were compared with published literature, and genomic subgroups were examined for prognostic impact. The median age at diagnosis was 70 years (range 24–92), with 68% de novo AML, 19% secondary AML, 10% therapy-related AML, and 3% APL. Of 126 treated patients, 72 received intensive therapy and 53 received less-intensive regimens. CR rates were 75% in the intensive cohort versus 36% in the less-intensive cohort. Karyotyping (available in 87%) revealed 47% with normal cytogenetics, favourable risk in 5% and 30% with adverse-risk features. Molecular profiling identified ELN 2022 adverse-, intermediate-, and favourable-risk categories in 42%, 47%, and 7% of intensively treated patients; in the less-intensive cohort, adverse risk predominated (87%). TP53 mutations occurred in 8% of the entire cohort. Median OS and PFS were 19 and 11 months, respectively. Younger age (<65 years), favourable / intermediate cytogenetics, de novo AML, favourable / intermediate-risk ELN 2022 classification, and TP53 wild-type status were associated with significantly superior survival (all *P* < 0.05). Intensive therapy conferred marked survival benefit (median OS 32 vs 9 months; HR 0.35, *P* < 0.0001). ELN 2024 risk groups did not discriminate survival in the less-intensive cohort. TP53 mutations were associated with significantly inferior OS (median 12 vs 23 months; *P* = 0.008). This single-centre study demonstrates a heterogeneous genomic landscape in Irish AML patients, with mutation frequencies broadly consistent with international datasets but with notable enrichment of adverse-risk disease in older and less-intensively treated populations. Integrated cytogenetic and molecular profiling strongly predicted outcomes, while age, treatment intensity, and TP53 status emerged as dominant prognostic factors. These findings highlight the critical value of comprehensive genomic assessment to guide stratified AML management within the Irish population.

## Introduction

Acute myeloid leukaemia (AML) is a genetically heterogeneous malignancy in which population-specific mutational patterns provide critical diagnostic, prognostic, and therapeutic insight [1–4]. Traditional cytogenetic analysis has long served as a cornerstone of AML risk assessment, defining favourable, intermediate, and adverse prognostic categories based on recurrent structural and numerical chromosomal abnormalities [5]. The introduction of high-throughput genomic sequencing has transformed AML classification, particularly among patients with cytogenetically normal AML, by uncovering pathogenic variants not detectable by karyotyping alone [2, 6–8]. This molecular revolution has expanded our understanding of leukemogenesis and accelerated development of targeted therapies.

Recurrent mutations in NPM1, FLT3, DNMT3A, IDH1/2, ASXL1, RUNX1, and TP53 have become central to diagnostic and prognostic models. Of these, NPM1 mutations (~ 30% of adult AML) and FLT3-ITD/TKD alterations (~ 25–30%) represent two of the most clinically impactful lesions, often occurring together and forming well-defined molecular subsets [1, 3, 7, 9–11]. Co-mutational patterns—such as NPM1–DNMT3A or NPM1–FLT3 combinations—are now recognised as biologically and prognostically significant and contribute to integrated genomic risk classification systems, including the 2022 and 2024 European Leukaemia Net (ELN) frameworks [6, 12, 13].

Despite advances in population-level AML genomics across Europe and North America, comprehensive cytogenetic–molecular characterisation of AML in Ireland remains limited. This study therefore evaluates the cytogenetic landscape, mutation spectrum, and clinical outcomes in a single-centre Irish AML cohort. We compare mutation frequencies with international datasets and assess associations between cytogenetic, molecular, and clinical prognostic factors providing real-world, population-specific insight into AML biology within the Irish healthcare setting.

## Methods

### Study design and patient cohort

This was a retrospective, single-centre study of adult patients diagnosed with AML at University Hospital Waterford between 2007 and 2025. Patients were identified through the departmental haematology database. Ethical approval for the study was obtained from the institutional review board. Inclusion criteria were age ≥ 18 years and diagnosis of AML according to WHO criteria. Patients with acute promyelocytic leukaemia (APL) were included for descriptive purposes but were excluded from some subtype-specific survival analyses as indicated.

### Data collection

Clinical data extracted from electronic and paper records included age at diagnosis, sex, AML subtype (de novo, secondary, therapy-related, APL), treatment modality (intensive versus less intensive therapy, or best supportive care), and treatment response. Response was assessed using standard criteria and categorised as complete response (CR), partial response (PR), refractory disease, or not evaluable (due to early death or intolerance).

### Cytogenetic and molecular analysis

Conventional karyotyping was performed on diagnostic bone marrow aspirates using standard G-banding techniques. Cytogenetic abnormalities were classified according to established risk categories (favourable, intermediate, adverse). Polymerase chain reactions (PCR) and Fluorescence in situ hybridisation (FISH) were used where appropriate to confirm specific alterations or rearrangements.

Since 2023, Targeted next-generation sequencing (NGS) panels were applied to diagnostic samples as they became available in routine practice. Genes analysed included commonly mutated loci in AML, such as NPM1, FLT3 (ITD and TKD), DNMT3A, TET2, IDH1, IDH2, RUNX1, ASXL1, SRSF2, NRAS, KRAS, STAG2, and TP53. Variant interpretation followed contemporary guidelines and local laboratory standards. Risk stratification was performed using the 2022 European Leukaemia Net (ELN 2022) classification in patients with sufficient molecular and cytogenetic data, particularly in the intensively treated cohort. ELN 2024 criteria were applied in a subset of patients treated with less-intensive regimens where data completeness permitted.

### Outcomes and statistical analysis

The primary endpoints were overall survival (OS), defined as time from diagnosis to death from any cause or last follow-up, and progression-free survival (PFS), defined as time from diagnosis to relapse, progression, or death. Secondary endpoints included response rates and the prognostic impact of age, AML subtype, cytogenetic risk, ELN risk classification, and TP53 mutational status. Survival analyses were conducted using GraphPad Prism (Version 10.06). Kaplan–Meier curves were generated and compared using the log-rank (Mantel–Cox) test. Hazard ratios (HRs) with 95% confidence intervals (CIs) were calculated. Mutation frequencies in this cohort were compared descriptively with those reported in the literature.

## Results

### Patient characteristics and treatment

A total of 165 adult patients with AML were diagnosed and managed at our centre during the study period (2007–2025). The median age at diagnosis was 70 years (range 24–92), with a male predominance (106 patients, 64%). The cohort comprised 117 patients (71%) with de novo AML including 4 patients (2%) with APL, 31 patients (19%) with secondary AML, 17 patients (10%) with therapy-related AML. 125 patients (76%) received AML-directed therapy, while 40 patients (24%) received best supportive care only. Among treated patients, intensive chemotherapy (Cohort 1) was administered to 72 patients (57%), and less-intensive therapy (Cohort 2) was used in 53 patients (43%). Details of induction regimens summarised in Table 1.

**Table 1:** Treatment of the study AML cohort

Intensive Therapy (72)	No = 72	%	Less-intensive therapy (53)	No = 53	%
DA 3 + 10	60	83%	**Hypomethylating Agents**	34	65%
Vyxeos	3	4%	**Hypomethylating Agents + BCL2i**	10	20%
FLAG-IDA	3	4%	**Hypomethylating Agents + FLT3i**	2	12%
DA 3 + 10+ GO	1	1%	**Low Dose Cytarabine**	6	12%
DA 3 + 10+FLT3 inhibitor	2	3%	**Lenalidomide**	1	2%
ATO+ATRA	3	4%			

### Treatment response

In Cohort 1, complete response (CR) was achieved in 54 of 72 patients (75%), while 16 patients (22%) had refractory disease (RD). Response was not documented in 2 patients (3%) due to early post-induction deaths. Among those achieving CR1, 10 patients (14%) underwent allogeneic stem cell transplantation (ASCT) consolidation. In Cohort 2, CR was achieved in 19 of 53 patients (36%) including 2 patients in this cohort proceeded to ASCT, partial response (PR) in 6 patients (11%), and refractory disease in 21 patients (40%). Treatment response was not assessable in 7 patients (13%) due to treatment intolerance or early death.

### Cytogenetic landscape

In the entire study cohort, conventional karyotyping was available for 145 of 165 patients (87%). Cytogenetic risk stratification identified: Favourable risk in 7 patients (5%), including 4 APL cases (3%) and 3 patients (2%) with inversion 16 alterations disease. Intermediate risk in 81 patients (56%), of whom 60 (41%) had a normal karyotype and adverse risk in 57 patients (40%).

### NPM1 & FLT3 mutations frequencies

NPM1 mutation status was available for 93 patients in the study cohort. Among these, 79 (85%) were NPM1-negative, whereas 14 (15%) were NPM1-positive; 5 of the NPM1-positive cases (5%) demonstrated co-occurring FLT3 mutations. FLT3 mutation screening was performed in 120 patients, identifying wild-type FLT3 in 100 cases and FLT3 mutations in 20 cases.

### Molecular profiling and ELN risk classification

Targeted NGS revealed a heterogeneous mutational landscape dominated by recurrent alterations in epigenetic regulators, splicing factors, signalling genes, and transcription factors. Molecular profiling was available for 69 of 72 intensively treated patients (96%). Applying ELN 2022 criteria in this group identified: Adverse risk: 30 (42%), Intermediate risk: 34 (47%) Favourable risk: 5 (7%).

**Fig. 1:**
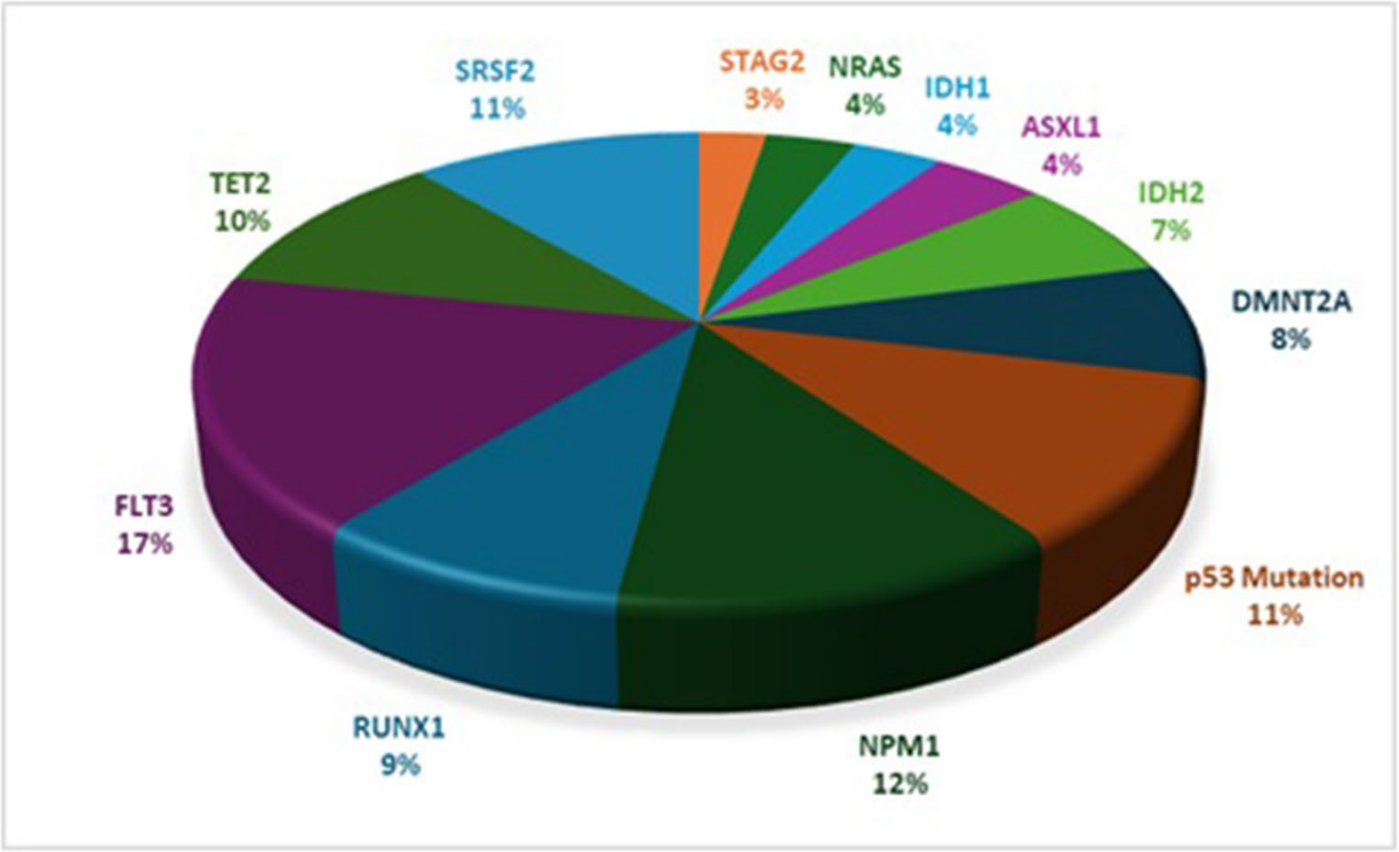
Frequency of the common NGS genetic alterations

**Fig. 2:**
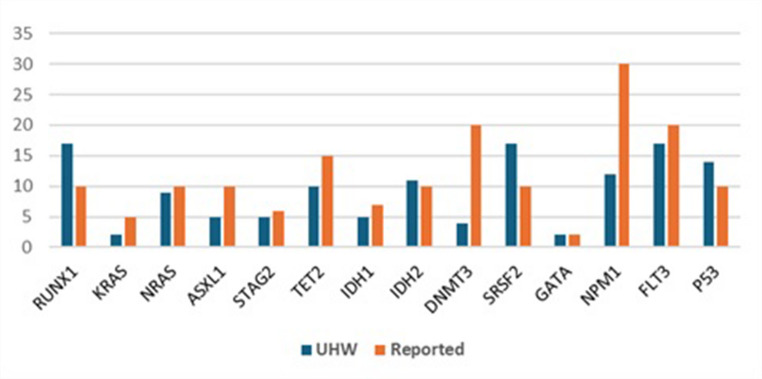
Frequency of NGS genetic alterations at UHW compared with reported data

In the less-intensive cohort, molecular data were available for 24 of 53 patients (45%). Based on ELN 2022 classification: Adverse risk: 21 (87%), Intermediate risk: 2(8%), Favourable risk: 1 (4%). Using ELN 2024 criteria in the less-intensive cohort, adverse risk was assigned to 5 patients and intermediate risk to 16 patients, reflecting the limited availability of complete molecular data in this group. Across the entire cohort, TP53 alterations were detected in 13 patients (11%). Figure 1 depicts the spectrum of recurrent genetic alterations identified in our AML study cohort, demonstrating a distribution comparable to that reported in previous studies (Fig. 2).

### Survival outcomes

**Fig. 3:**
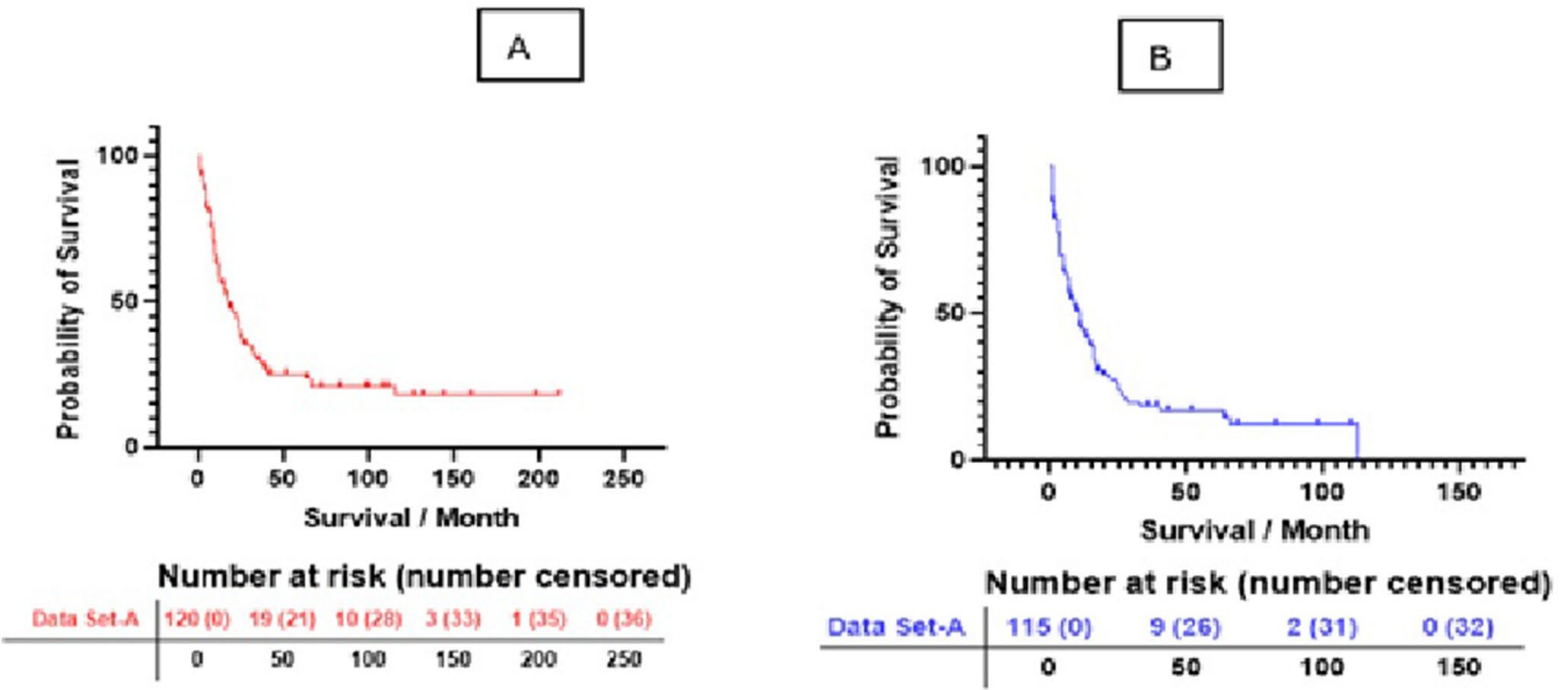
OS (**A**) & PFS (**B**) KM curves treated AML cohort

For the entire treated AML cohort (intensive & less intensive), the median OS was 19.0 months, and the median PFS was 11.0 months. This indicates that half of treated patients survived beyond approximately 1.5 years after therapy initiation and that half experienced progression or death within about one year (Fig. 3).

**Fig. 4:**
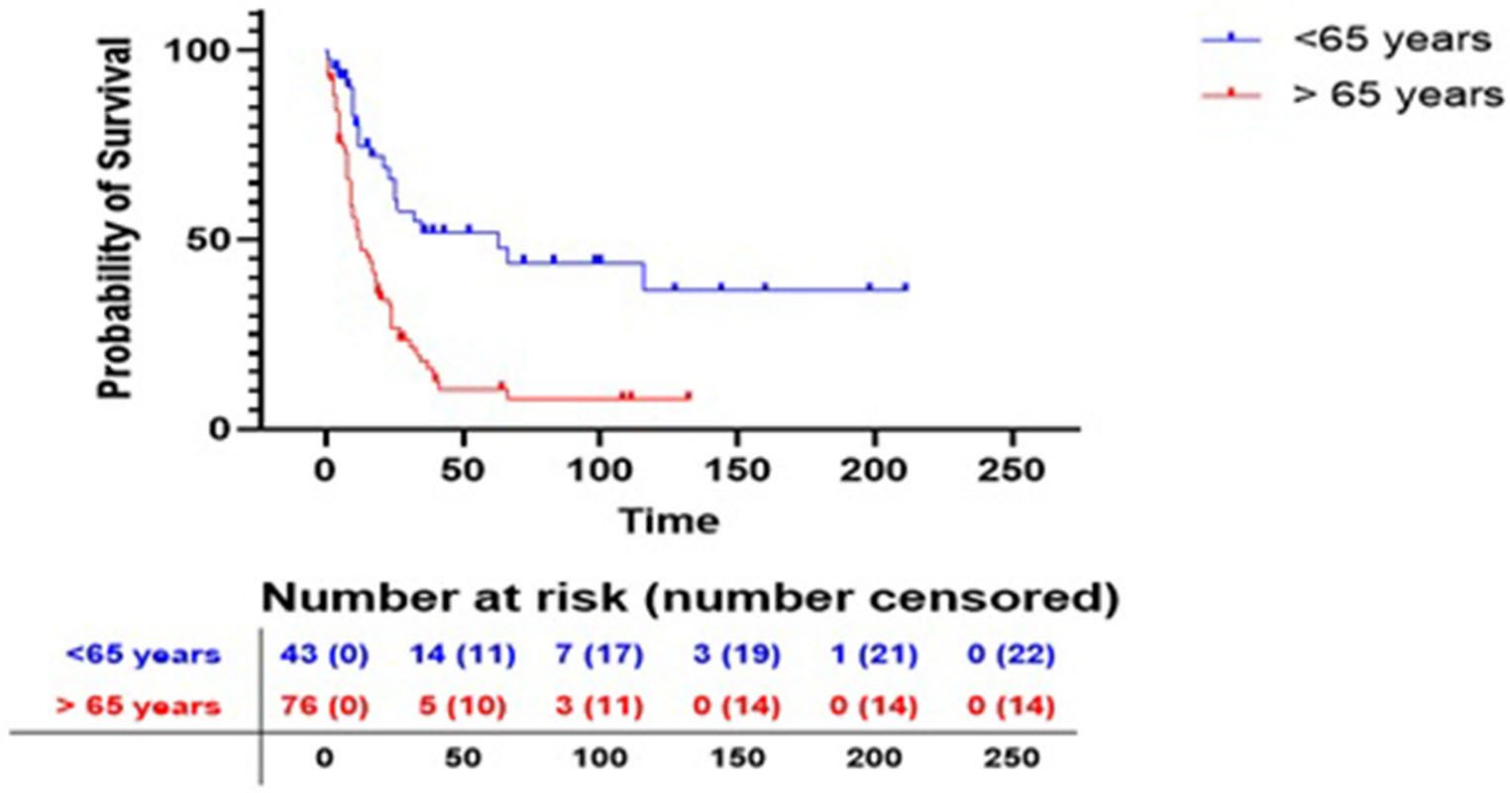
OS KM curves for treated AML cohort per Age

Age was a strong predictor of OS. Patients younger than 65 years had significantly better survival than those aged ≥ 65 years (log-rank *P* < 0.0001). Median OS was 63 months in the < 65-year group compared with 13 months in the ≥ 65-year group. The HR for death was 0.3682 (95% CI: 0.2393–0.5663), indicating approximately a 63% reduction in mortality risk in younger patients (Fig. 4).

**Fig. 5:**
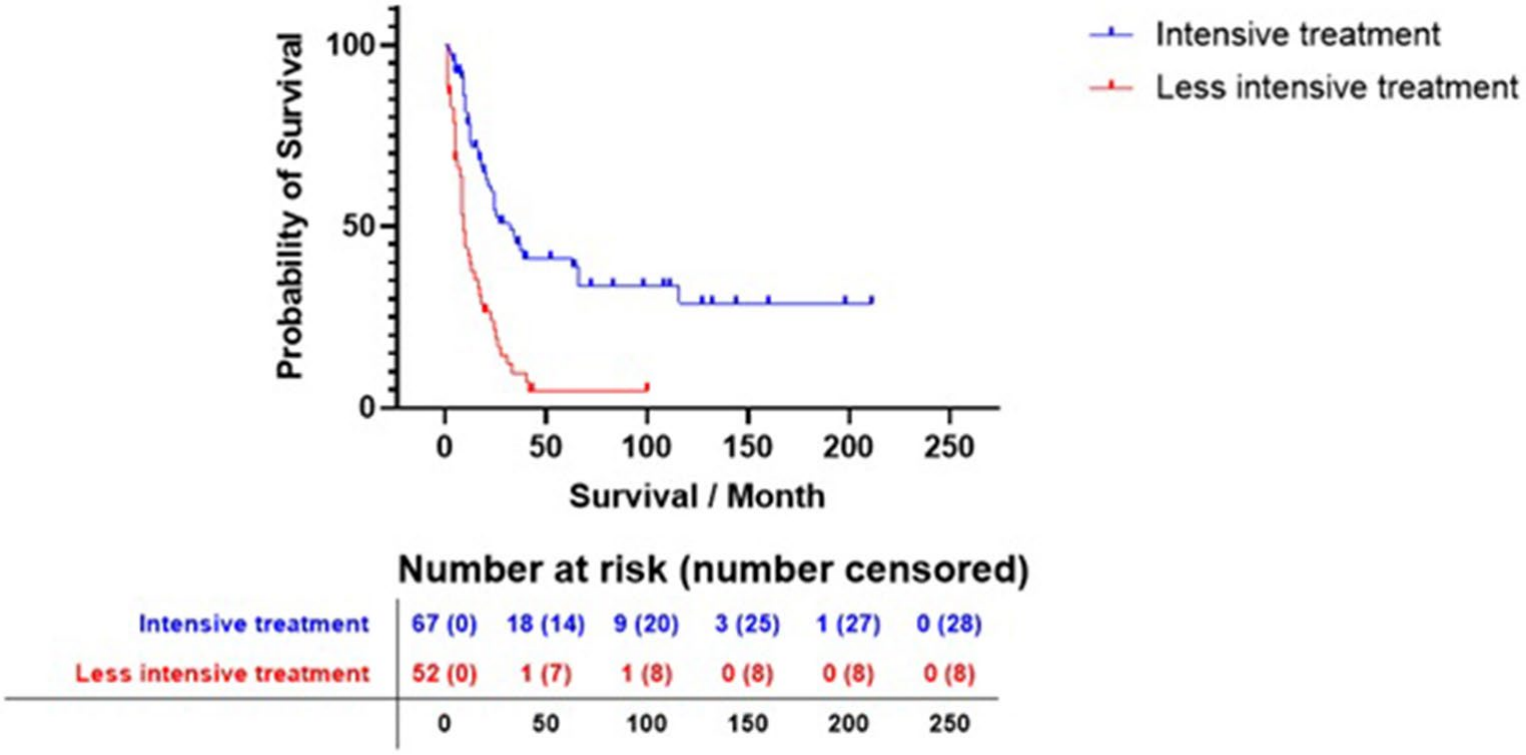
OS KM curves for AML cohort per treatment sub-type

Intensive therapy was associated with significantly improved OS compared with less-intensive therapy (log-rank *P* < 0.0001). The hazard ratio for death was 0.3488 (95% CI: 0.2163–0.5626), indicating a markedly lower mortality risk in the intensive treatment group. Median OS was 32 months in intensively treated patients versus 9 months in those receiving less-intensive regimens. Kaplan–Meier curves demonstrated a clear and sustained survival advantage for intensive therapy over the follow-up period. (Figure 5) Cytogenetic risk stratification correlated significantly with OS (log-rank *P* = 0.0178).

**Fig. 6:**
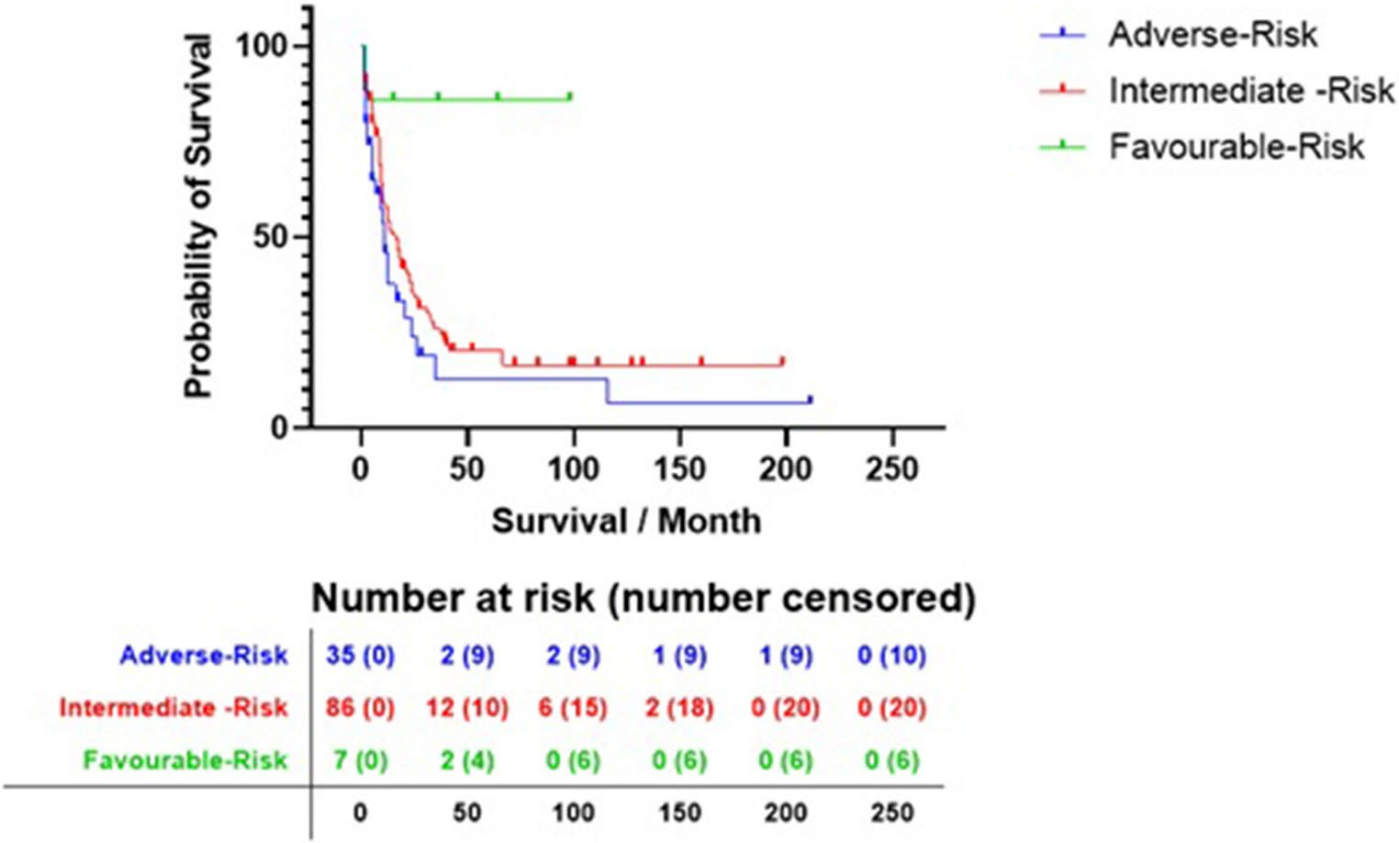
OS KM curves for treated AML cohort per cytogenetic risk

Median OS was: 11 months for adverse-risk cytogenetics, 16 months for intermediate-risk cytogenetics and not reached for favourable-risk cytogenetics. Favourable-risk patients demonstrated sustained long-term survival, whereas adverse-risk patients experienced the most rapid early decline (Fig. 6).

**Fig. 7:**
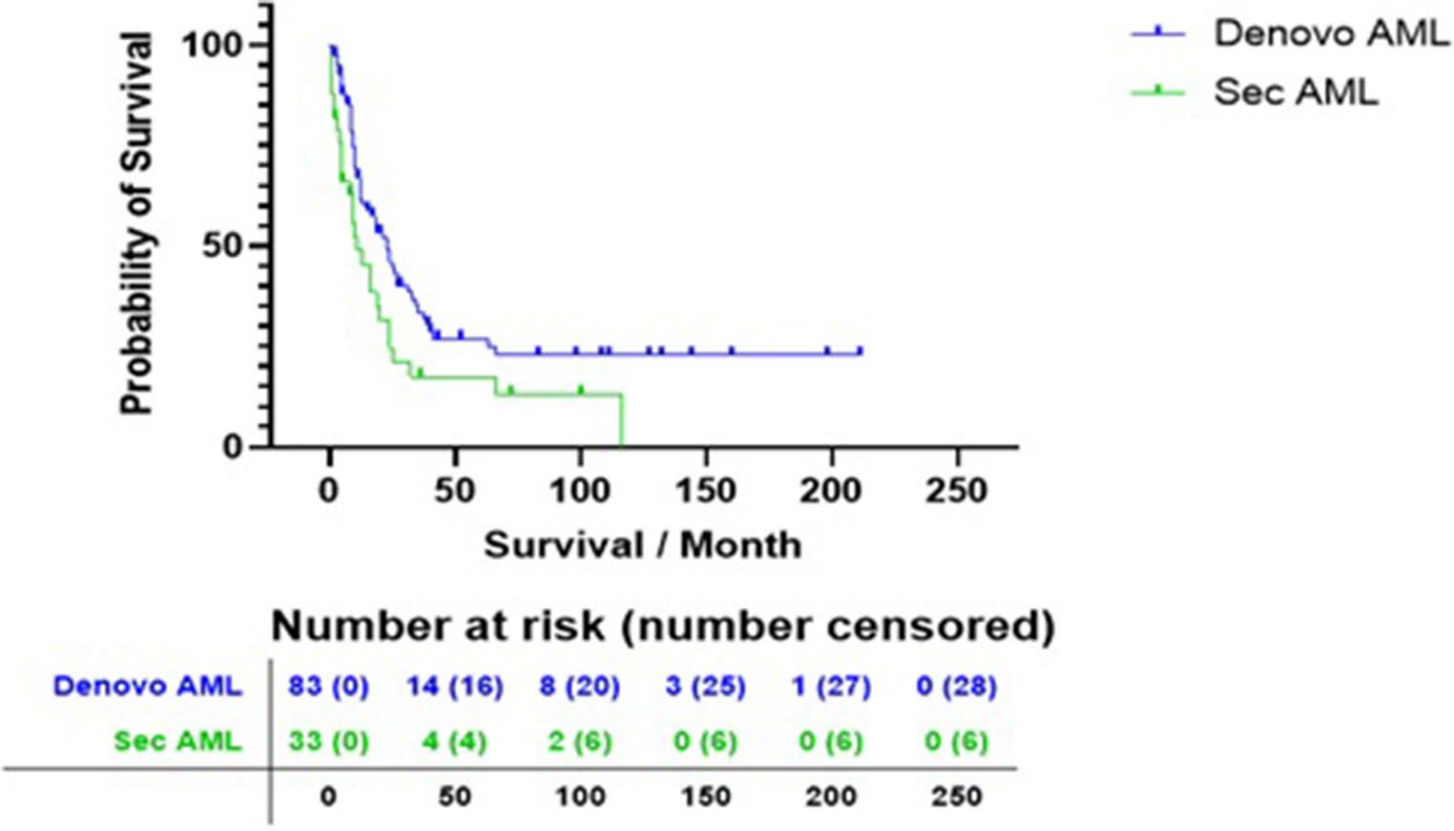
OS KM curves for treated AML cohort per disease sub-type

Excluding APL, patients with de novo AML had significantly better OS than those with secondary AML (log-rank *P* = 0.0193). Median OS was 23 months for de novo AML and 11 months for secondary AML (post MDS/MPN & Therapy related AML). The HR for death in secondary AML versus de novo disease was 0.5871 (95% CI: 0.3493–0.9869), indicating nearly double the mortality risk in secondary AML (Fig. 7).

**Fig. 8:**
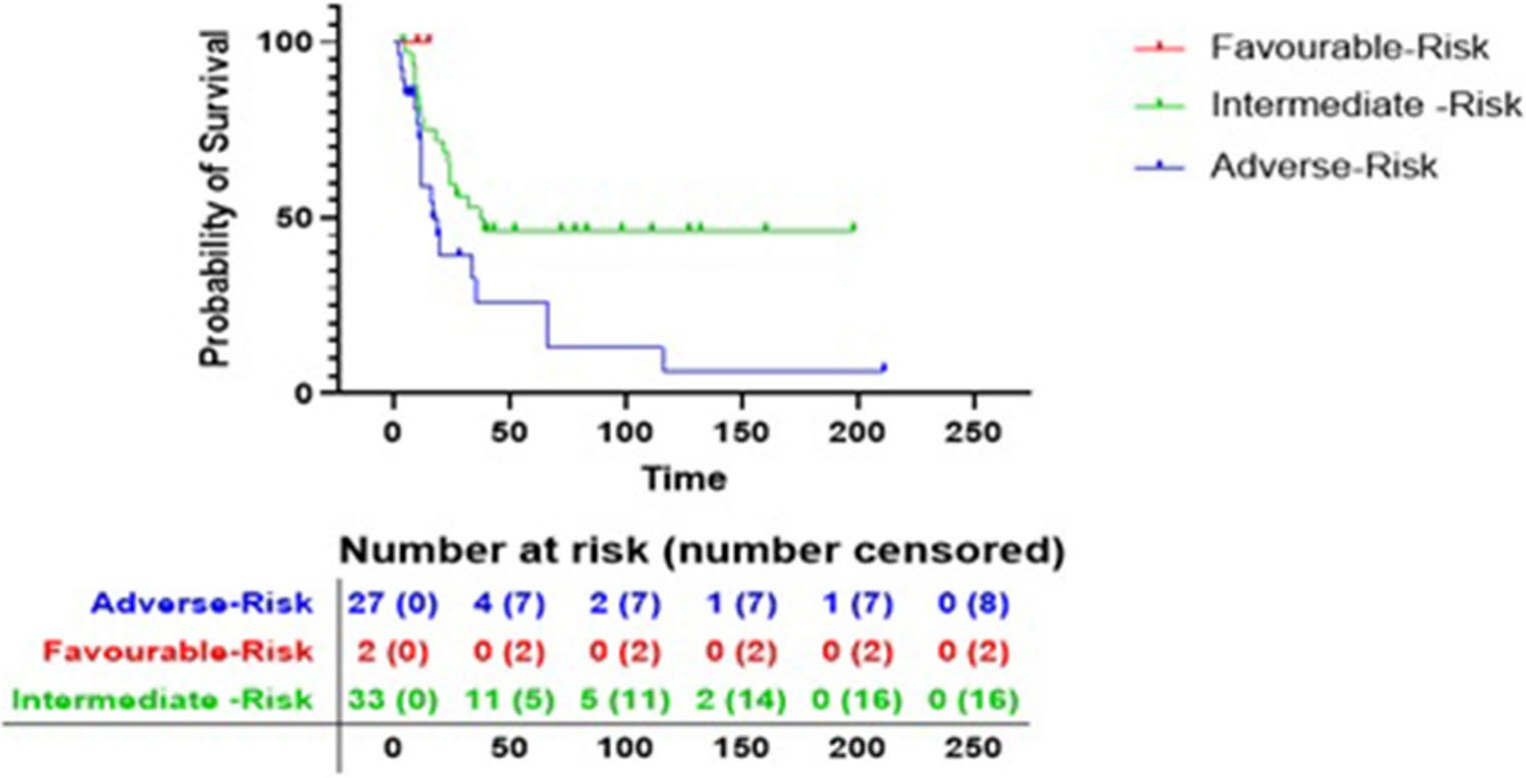
OS KM curves for treated AML cohort per ELN 2022 risk stratification

In the intensively treated AML cohort (excluding APL cases), the ELN 2022 genetic risk classification demonstrated significant prognostic value. Overall survival differed markedly across risk groups (log-rank *P* = 0.0324). Patients in the adverse-risk category had the poorest outcomes, with a median OS of 17 months and a steep early decline in survival. The intermediate-risk group achieved the most favourable long-term outcomes, with a median OS of 37 months and a sustained survival plateau extending beyond 150–200 months. The favourable-risk group showed encouraging early survival and did not reach a median OS, although the small sample size (*n* = 2) limits definitive interpretation. These findings confirm the discriminatory power of ELN 2022 classification in predicting outcomes among AML patients receiving intensive chemotherapy [1] (Fig. 8).

In the less-intensive therapy cohort, ELN 2024 risk categories did not significantly differentiate OS (log-rank *P* = 0.4929). Median OS was 7 months in the adverse-risk group, 8 months in the intermediate-risk group, and 12 months in the favourable-risk group, indicating limited discriminatory power of ELN risk categories in this frailer, less intensively treated population.

**Fig. 9:**
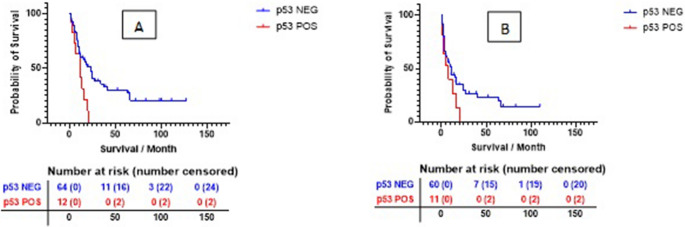
OS (**A**) & PFS (**B**) KM curves for treated AML cohort per p53 mutational status

TP53 (p53) mutational status significantly stratified OS (log-rank *P* = 0.0080). Median OS was 23 months in p53-negative patients and 12 months in p53-mutated patients. The HR for death was 0.4164 (95% CI: 0.1613–1.075), indicating a strong trend toward increased mortality in TP53-mutated AML. Kaplan–Meier curves showed a striking early decline in survival for p53-positive patients. For PFS, there was a trend toward inferior outcomes in p53-mutated AML (log-rank *P* = 0.0914). Median PFS was 11 months in p53-negative versus 8 months in p53-positive patients (HR 0.5578, 95% CI: 0.2292–1.358), with earlier separation of curves in the TP53-mutated group (Fig. 9).

## Discussion

This single-centre study provides a detailed description of the clinical, cytogenetic, and molecular landscape of AML in an Irish population over nearly two decades. The age distribution, predominance of de novo AML, and high proportion of older patients mirror contemporary “real-world” AML cohorts.

Age, cytogenetic risk, ELN classification, disease subtype, and TP53 status all demonstrated strong prognostic influence. Younger age, favourable or intermediate cytogenetics, de novo AML, favourable / intermediate ELN 2022 risk (in intensively treated patients), and TP53 wild-type status were associated with superior outcomes. In contrast, older age, adverse cytogenetics, secondary AML, adverse ELN 2022 risk, and TP53 mutation consistently identified high-risk groups. Such finding aligns with international AML genomic landscapes [1–4, 6–12, 17], but with expected differences attributable to age structure, secondary AML prevalence, and regional referral patterns [4, 18].

These results comparable with international experience and consistent with published global data [6, 18–22]. This confirms the applicability of integrated molecular–cytogenetic risk models to the Irish AML population.

Limited discriminatory power of ELN 2024 in the less-intensively treated cohort parallels experience in older/frail populations where biology and treatment constraints intersect [13]. This limitation may reflect the predominant older patient’s cohort included and use of hypomethylating agent monotherapy with the minimal exposure to BCL2 inhibition within the study cohort which underscores the need for refined prognostic tools and novel therapeutic options in this setting.

Distribution of cytogenetic abnormalities was broadly consistent with large international AML datasets [5, 6, 17] illustrated in Table 2. A relative enrichment of adverse cytogenetics particularly complex karyotypes is typical in older real-world cohorts and those enriched for secondary AML [4, 18]. This may reflect an older or more secondary-AML-enriched population.

**Table 2:** Cytogenetic risk stratification compared with reported literature

Risk Group	UHW Cohort	Typical Published Range	Interpretation
Favourable	5%	10–15%	Lower than expected
Intermediate	56%	40–50%	Slightly higher but consistent
Normal karyotype subset	41%	40–50%	Well-aligned
Adverse	40%	20–30%	Higher than expected

Observed NPM1 (15%) and FLT3 (17%) rates were lower than widely reported values (~ 30% and 25–30%, respectively) [1, 3, 7, 9]. Co-occurrence patterns mirrored known genomic subsets [1, 3, 15]. Co-mutation analysis highlighted recurrent NPM1–DNMT3A and FLT3-ITD–NPM1 combinations, which mapped onto different ELN risk strata.

This may reflect institutional referral patterns as historically high risk fit young patients were referred at diagnosis to transplant centre for induction and transplant consideration as appropriate, in addition more elderly AML cases in this cohort may contributing to such differences.

NGS revealed expected enrichment of mutations in DNMT3A, TET2, ASXL1, SRSF2, and signalling genes typical of mixed de novo/secondary AML cohorts [8, 11, 16–18]. The adverse impact of TP53 alterations aligns with established high-risk biology [19–21]. This pattern is consistent with recognised AML biology, with frequent involvement of epigenetic modifiers (TET2, DNMT3A, ASXL1), splicing factors (SRSF2), and signalling genes (FLT3, NRAS, KRAS). Adverse-risk molecular features—including TP53, RUNX1, ASXL1, and SRSF2 mutations—were more common in older and less-intensively treated patients, as observed in other real-world cohorts [18, 19, 22].

The presence of TP53 mutations in a notable subset underscores a population with high-risk molecular features. The functional category analysis underscores the central roles of dysregulated signalling, impaired DNA damage repair (particularly TP53), and epigenetic deregulation in driving disease pathogenesis. The relative enrichment of complex karyotypes and the burden of adverse molecular features highlight the clinical challenge of managing high-risk AML in routine practice [1–4].

Figure 2 illustrate the distribution of key genetic alterations and compare their frequencies with those reported in the literature. Overall, our AML cohort mutations profile diverges from published cohorts by showing a lower prevalence of typical de novo AML mutations and a higher prevalence of mutations associated with secondary or high-risk disease. These differences may reflect sample size, age structure, referral patterns, or regional biology but do not detract from overall concordance with established AML mutation landscapes.

Limitations of this study include its retrospective design, single-centre nature, and incomplete molecular profiling in earlier years of the study period and in some less-intensively treated patients. Nonetheless, the cohort provides valuable “real-world” data from a defined geographical population and offers a foundation for future collaborative Irish or international studies.

## Conclusion

This study demonstrates that the genomic landscape of AML in an Irish single-centre cohort is broadly comparable to that reported internationally, with a heterogeneous mix of favourable and adverse molecular features. Integrated cytogenetic and molecular profiling, interpreted through ELN risk frameworks, robustly stratifies prognosis, while age, disease subtype, treatment intensity, and TP53 status emerge as dominant determinants of outcome.

Our findings emphasise the importance of comprehensive genomic assessment in routine AML care, support the applicability of current risk models to the Irish setting, and highlight the unmet need for improved therapeutic strategies for older patients, those receiving less-intensive therapy, and patients with adverse-risk or TP53-mutated disease.

## Data Availability

No datasets were generated or analysed during the current study.
